# Towards personalized medicine by mathematical modeling of tumors

**DOI:** 10.12688/openreseurope.14814.1

**Published:** 2022-05-18

**Authors:** Dániel András Drexler, Levente Kovács, Gheorghe Moza

**Affiliations:** 1 John von Neumann Faculty of Informatics, Obuda University, Budapest, Hungary; 2 Department of Mathematics, Politehnica University of Timisoara, Timisoara, Romania

**Keywords:** mathematical modeling; cancer models

## Abstract

In this open letter, we give some insights on the potential use of mathematical modeling in understanding cancer research. The article is written in a form that can be understood by a larger public, not only by specialists.

## On applications of mathematical modeling

In the modern world, cancer is one of the leading causes of death, with almost 2 million new cases and 600,000 deaths in the USA alone in 2020 ^1^. Still, intensive research is carried out to treat tumor cells, starting from the classical chemotherapy, towards more specific therapies, like targeted molecular therapies and immunotherapies. Many oncologists believe that classical chemotherapies have reached their maximum efficiency and cannot be further enhanced. Although, other researchers do not agree with this.

The current chemotherapy protocols are based on the maximal tolerable dose (MTD), which is injected with a predefined schedule, attacking the tumor and the body of the patient aggressively like “carpet bombing”. A promising new direction of therapy development is optimization of the protocol, that is, use as large dose as necessary, at the time it is necessary. However, it is very hard to identify the necessary dose and time of injection. This is where an approach based on mathematical modeling comes into the picture ^2^.

The application of mathematical models in clinical practice relies on strong collaboration with medical experts who have limited knowledge on mathematical modeling and, particularly, the theory of differential equations. The cooperation can be boosted if we describe the mathematical laws (such as equations and constraint conditions) in the way they can be interpreted by biologists as well. Our research group at the Physiological Controls Research Center of Obuda University uses formal reaction kinetics analogy ^3^ and ^4^ to interpret tumor modeling for experts not familiar with differential equations.

Although a mathematical model is fundamental for the optimization of drug therapies, it is only a first step towards personalized medicine. There are many issues that need to be solved. The first one is the identification of the model parameters, which gives rise to several problems such as:

1. Since in experiments we can only measure the total tumor volume and have limited number of measurements, the parameter set describing the behavior is not unique; there are infinitely many parameter sets such that the model gives the same output.
2. The parameters change over time, since the tumor adapts to the treatment, and the patient is also affected by the chemotherapy.
3. The system is impulsive, that is, the inputs are injections, and they introduce discontinuities in the model.

On the other hand, these problems give rise to interesting new approaches, like application of artificial intelligence and nonlinear optimization algorithms for the physiological problem. Finding the optimal set of injections is also challenging, due to the limited measurements on the system and the inaccuracies in the parametric identification, and also by the impulsive nature of the system. Application of nonlinear optimization techniques shows a potential way towards personalized treatment, however, increasing robustness is still subject to further research.

Mathematical modeling in biology and, particularly, in cancer research has the potential of reducing considerably the costs of the research, which usually arise from various laboratory-based experiments. Once the biological laws of the disease’s evolution are known up to a good extent, they often can be described by theoretical mathematical models, based on a variety of methods and tools. The analysis of these models contributes, in turn, to a better understanding of the disease, thus, new opportunities for finding solutions to these abnormalities may arise.

In conclusion, the optimization based on mathematical modeling is a promising direction towards personalized medicine. The first step is creating relevant mathematical models of the problem, which need to be validated by experiments. However, after this first step, there is still a long way to go until personalized medicine can be used in clinical practice.

## 2 Data availability

No data are associated with this article.
