# Supplementary material for: Towards personalized medicine by mathematical modeling of tumors
Source: Open Res Eur. 2022 May 18;2:59. [Version 1] doi: 10.12688/openreseurope.14814.1 (PMC10446033; doi:10.12688/openreseurope.14814.1)
Supplement: Peer review history

# Peer review: Reviewer response for version 1

**DOI:** 10.21956/openreseurope.16000.r29304

**Published:** 2022-06-28

**Authors:** Adriana Buică

**Affiliations:** 1 Department of Mathematics, Babeș-Bolyai University, Cluj-Napoca, Romania

This article supports the use of mathematical modeling of tumors with the aim of introducing a personalized treatment of cancer. This is a noble and ambitious target and, as the authors also emphasized, difficult to realize. Based on their experience, they proposed the use of differential equations, artificial intelligence, and nonlinear optimization algorithms. They also discuss issues on the challenges of the cooperation between the medical experts and the mathematicians.

Where applicable, are recommendations and next steps explained clearly for others to follow? (Please consider whether others in the research community would be able to implement guidelines or recommendations and/or constructively engage in the debate)

Yes

Does the article adequately reference differing views and opinions?

Yes

Are all factual statements correct, and are statements and arguments made adequately supported by citations?

Yes

Is the rationale for the Open Letter provided in sufficient detail? (Please consider whether existing challenges in the field are outlined clearly and whether the purpose of the letter is explained)

Yes

Is the Open Letter written in accessible language? (Please consider whether all subject-specific terms, concepts and abbreviations are explained)

Yes

Reviewer Expertise:

mathematics (differential equations)

I confirm that I have read this submission and believe that I have an appropriate level of expertise to confirm that it is of an acceptable scientific standard.

# Peer review: Reviewer response for version 1

**DOI:** 10.21956/openreseurope.16000.r29302

**Published:** 2022-05-20

**Authors:** Andrea De Gaetano

**Affiliations:** 1 Istituto di Analisi dei Sistemi ed Informatica “Antonio Ruberti” (CNR-IASI), Rome, Italy

This is a letter directed to the general public, and its language is appropriate for the scope.

 My only doubt refers to the introduction of artificial intelligence or similar techniques as an alternative modelling approach right after having stated (correctly) that data insufficiency often prevents (structural) model identification. It is not clear how artificial intelligence would improve the situation.

Where applicable, are recommendations and next steps explained clearly for others to follow? (Please consider whether others in the research community would be able to implement guidelines or recommendations and/or constructively engage in the debate)

Yes

Does the article adequately reference differing views and opinions?

Partly

Are all factual statements correct, and are statements and arguments made adequately supported by citations?

Yes

Is the rationale for the Open Letter provided in sufficient detail? (Please consider whether existing challenges in the field are outlined clearly and whether the purpose of the letter is explained)

Yes

Is the Open Letter written in accessible language? (Please consider whether all subject-specific terms, concepts and abbreviations are explained)

Yes

Reviewer Expertise:

mathematical modelling in biomedicine

I confirm that I have read this submission and believe that I have an appropriate level of expertise to confirm that it is of an acceptable scientific standard.
